# Individual markers of cerebral small vessel disease and domain‐specific quality of life deficits

**DOI:** 10.1002/brb3.2106

**Published:** 2021-03-10

**Authors:** Jeevan Fernando, Robin B. Brown, Hayley Edwards, Marco Egle, Hugh S. Markus, Jonathan Tay

**Affiliations:** ^1^ Stroke Research Group Department of Clinical Neurosciences University of Cambridge Cambridge UK

**Keywords:** cerebral small vessel disease, executive function, functional disability, lacunar infarcts, quality of life, stroke

## Abstract

**Background:**

Cerebral small vessel disease (SVD) leads to reduced quality of life (QOL), but the mechanisms underlying this relationship remain unknown. This study investigated multivariate relationships between radiological markers of SVD and domain‐specific QOL deficits, as well as potential mediators, in patients with SVD.

**Methods:**

Clinical and neuroimaging measures were obtained from a pooled sample of 174 SVD patients from the St. George's Cognition and Neuroimaging in Stroke and PRESsure in established cERebral small VEssel disease studies. Lacunes, white matter hyperintensities, and microbleeds were defined as radiological markers of SVD and delineated using MRI. QOL was assessed using the Stroke‐Specific Quality of Life Scale. Multivariate linear regression was used to determine whether SVD markers were associated with domain‐specific QOL deficits. Significant associations were further investigated using mediation analysis to examine whether functional disability or cognition was potential mediators.

**Results:**

Multivariate regression analyses revealed that lacunes were associated with total QOL score (*β* = −8.22, *p* = .02), as well as reductions in mobility (*β* = −1.41, *p* = .008) and language‐related subdomains (*β* = −0.69, *p* = .033). White matter hyperintensities and microbleeds showed univariate correlations with QOL, but these became nonsignificant during multivariate analyses. Mediation analyses revealed that functional disability, defined as reduced activities of daily living, and executive function, partially mediated the relationship between lacunes and total QOL, as well as mobility‐related QOL, but not language‐related QOL.

**Conclusions:**

Lacunar infarcts have the most detrimental effect on QOL in SVD patients, particularly in the mobility and language‐related subdomains. These effects may be partially explained by a reduction in activities of daily living. These results may inform targeted interventions to improve QOL in patients with SVD.

## Graphical abstract

Fernando et al. investigate associations between radiological markers of cerebral small vessel disease (SVD) and subdomains of quality of life (QOL). In a pooled sample of SVD patients, they find that lacunes lead to reductions in total QOL, as well as mobility and language‐related QOL, and explore potential mediating variables. These results suggest the possibility of targeted interventions to improve QOL in SVD patients.

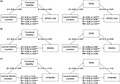

## 1 INTRODUCTION

Cerebral small vessel disease (SVD) affects the small vessels of the brain, leading to stroke, cognitive impairment, and mortality (van Leijsen et al., 2019; Pantoni, 2010; Tuladhar et al., 2020). SVD has a substantial individual impact, causing functional disability and neuropsychiatric symptoms such as apathy and depression, which lead to subsequent reductions in quality of life (QOL) (Brookes et al., 2014; Tay et al., 2021).

Quality of life refers to an individual's sense of well‐being, which is recognized as an important health outcome (Wilson & Cleary, 1995). QOL can be divided into subdomains reflecting different aspects of physical and mental health (Williams et al., 1999). Despite its importance, little is known about the relationships between radiological markers of SVD and QOL subdomains, precluding interventions targeting specific aspects of QOL.

Our study had two objectives. First, we investigated the relationships between radiological markers of SVD and individual subdomains of QOL. Second, we attempted to elucidate the factors that mediated these relationships. Based on previous research (Brookes et al., 2014), we hypothesized that SVD pathology would be associated with mobility and mood related deficits and that these associations could be partially explained by deficits in functional abilities or executive function.

## 2 METHODS

### 2.1 Participants

Participants were symptomatic SVD patients from two studies: the St. George's Cognition and Neuroimaging in Stroke (SCANS) study and the “How intensively should we treat blood PRESsure in established cERebral small VEssel disease?” (PRESERVE) study (Croall et al., 2018; Lawrence et al., 2013). SCANS was a prospective cohort study of SVD patients, while PRESERVE was a multi‐center randomized controlled trial investigating intensive blood pressure control in hypertensive SVD patients. Both studies defined SVD as a clinical lacunar stroke syndrome with a corresponding lacunar infarct and confluent WMH of Fazekas grade ≥2 on MRI (Bamford et al., 1991; Fazekas et al., 1987). Patients with other types of stroke were excluded, including those with: cerebral artery stenosis >50%, cardioembolism, infarct diameter >1.5 cm, or a cortical infarct. Participants underwent MRI and neurocognitive assessments a minimum of 3 months after the most recent stroke to minimize the effect of the acute infarct on outcomes. Full details on each cohort can be found elsewhere (Croall et al., 2018; Lawrence et al., 2013).

### 2.2 Neuroimaging

#### 2.2.1 MRI acquisition

Images were acquired using 1.5T and 3T MRI in SCANS and PRESERVE, respectively. Both studies obtained a three‐dimensional T1‐weighted (T1w) image and a T2‐weighted (T2w) fluid attenuated inversion recovery (FLAIR) image. Full acquisition protocols have been detailed elsewhere (Croall et al., 2018; Lawrence et al., 2013).

#### 2.2.2 Radiological markers of SVD

Both datasets used identical definitions of radiological markers of SVD. Lacunes were defined as CSF‐filled cavities ≥3 mm in diameter and delineated using T1w and FLAIR images. WMH were regions of high signal intensity on FLAIR images and delineated using a semiautomated pipeline in SCANS semiautomatic contouring technique using the Jim image analysis software (Xinapse Systems Limited) in PRESERVE (Croall et al., 2018; Lambert et al., 2016). WMH segmentations were inspected and manually corrected where necessary. Microbleeds were defined as focal areas of low signal on T2w scans with a diameter <10 mm.

### 2.3 Clinical measures

#### 2.3.1 Quality of life

The Stroke‐Specific Quality of Life Scale (SSQOL) was used to measure patient QOL in SCANS and PRESERVE (Williams et al., 1999). The SSQOL contains 49 items assessed on a 5‐point Likert scale, with higher scores indicating better QOL. Individual items can be grouped into 12 subdomains of QOL: energy, family roles, language, mobility, mood, personality, self‐care, social roles, thinking, upper extremity function, vision, and work/productivity.

#### 2.3.2 Functional disability

Functional disability was defined as impairment in basic and instrumental activities of daily living (ADLs). Basic ADLs reflect a patient's independence in completing tasks such as feeding and bathing. Instrumental ADLs reflect more complex tasks that require planning.

SCANS used the Barthel Index and the Instrumental ADL scale to measure basic and instrumental ADLs, respectively, whereas the Disability Assessment for Dementia Scale was used to derive basic and instrumental ADL scores in PRESERVE (Gélinas et al., 1999; Lawton & Brody, 1969; Mahoney & Barthel, 1965). To ensure that measures of functional disability were consistent across datasets, we converted raw totals for all scores into percentages, thereby normalizing the scale and range. Density plots revealed the underlying distributions were relatively similar (Figure S1).

#### 2.3.3 Cognitive function

Cognition was assessed using two tests of executive function: the Trail Making Test B (TMTB) and verbal fluency (VF). The TMTB requires one to make a trail between alternating numbers and letters, with shorter times reflecting better cognitive function. VF requires one to name as many words starting with a certain letter of the alphabet, with higher scores reflecting better cognitive function. Raw scores on both tests were transformed into *z*‐scores using age and education‐based normative data. Full details on the tests and scoring have been published elsewhere (Lawrence et al., 2013).

### 2.4 Statistical analysis

Key variables were compared between SCANS and PRESERVE using Welch *t* tests, Wilcoxon rank‐sum tests, and chi‐square tests where appropriate. Given similar sample characteristics, participants in SCANS and PRESERVE were pooled for further analysis. As PRESERVE was a multi‐center trial, participants from SCANS were modeled as a different study site in PRESERVE. Using this study site variable as a covariate therefore controlled for variance from participants in SCANS in addition to the variance from multiple study sites in PRESERVE.

We first assessed univariate correlations between radiological markers of SVD and QOL subdomains using Pearson's or Spearman's correlations where appropriate. First, we attempted to assess univariate associations between radiological markers of SVD and QOL subdomains, which was done using Pearson's or Spearman's correlations where appropriate. Significant univariate associations were carried forward to multivariate linear regression analyses, which used the significant QOL subdomain as the dependent variable and the SVD markers as independent variables. Age, sex, National Adult Reading Test (NART) score, ethnicity, and study site were included as covariates in multivariate analyses.

Significant multivariate associations between SVD markers and QOL subdomains were then carried forward to causal mediation analysis, a statistical technique used to determine whether variance in the relationship between two variables can be explained by a third mediating variable (Imai & Yamamoto, 2013). These were conducted to identify potential variables that may explain significant multivariate associations between SVD markers and QOL domains. Based on previous research, we *a priori* chose functional disability and measures of executive function (TMTB, VF) as potential mediating variables (Brookes et al., 2014).

Statistical analyses were conducted using R 3.6.1 and the “mediation” package 4.4.7.17 (Imai & Yamamoto, 2013; R Core Team, 2019). All tests were two‐tailed with *α* = 0.05.

### 2.5 Ethical statement

SCANS received ethical approval from the London–Wandsworth ethics committee (ukctg.nihr.ac.uk; study ID: 4577). PRESERVE received ethical approval from the Harrow National Research Ethics Service committee (REC number: 11/LO/0458) and is registered with the U.K. Clinical Research Network (CRN number: 10962). All participants provided written informed consent according to the Declaration of Helsinki.

## 3 RESULTS

### 3.1 Sample characteristics

Of the 121 participants recruited in SCANS, 94 had complete data. Of the 167 participants recruited in PRESERVE, 80 had complete data. Both cohorts were similar on key demographic and outcome variables, showing only minor differences with regard to vascular risk factors and lacune count (Table 1). Due to these similarities, participants from SCANS and PRESERVE were pooled for subsequent analyses.

**TABLE 1:** Demographic and clinical characteristics of patients from SCANS and PRESERVE used in the analysis

	SCANS (*n* = 94)	PRESERVE (*n* = 80)	*p* value
Age	69.1 (10.0)	68.5 (9.2)	.653
Sex, Female (%)	25 (26.6)	33 (41.3)	.060
NART	100.2 (15.5)	116.1 (7.8)	**<.001**
Ethnicity			
Caucasian (%)	70 (74.5)	70 (88.6)^a^	**.029**
Black (%)	21 (22.3)	6 (7.6)	
Asian (%)	3 (3.2)	3 (3.8)	
Hypertension (%)	87 (92.6)	80 (100.0)	**.015**
Smoking			
Never (%)	31 (33.0)	39 (49.4)^a^	**.008**
Ex (%)	44 (46.8)	26 (32.9)	
Current (%)	19 (20.2)	14 (17.7)	
Diabetes (%)	15 (16.0)	17 (21.3)	.492
Hypercholesterolemia (%)	81 (86.2)	70 (87.5)	.973
Lacunes	4.1 (5.7)	4.9 (5.1)	**.025**
WMH	37,290 (27,110)	35,630 (23,610)	.855
Cerebral microbleeds	5.2 (18.5)	4.6 (8.1)	.088
TMTB	−1.13 (1.63)	−0.90 (1.56)	.385
Verbal Fluency	−0.25 (1.37)	−0.36 (1.23)	.817
Basic ADLs	97.7 (5.4)	91.8 (17.4)	.175
Instrumental ADLs	93.9 (13.2)	89.2 (20.3)	.095

Abbreviations: ADL, activities of daily living; NART, national adult reading test; TMTB, trail making test B; WMH, white matter hyperintensity volume.

^a^ Missing data (*n* = 1).

### 3.2 Relationships between radiological markers of SVD and QOL

Lacunes, WMH, and microbleeds were correlated with total SSQOL score, as well as several QOL subdomains (Table 2). Multiple regression models were then used to further analyze the relationships between SVD markers and QOL domains while controlling for age, sex, NART, ethnicity, study site, and other SVD markers. Results revealed that, after controlling for the effects of demographic variables and other SVD markers, associations only remained between lacunes and total SSQOL, as well as mobility and language subdomains (Table 3). No other SVD marker was associated with QOL subdomains in multivariate analyses.

**TABLE 2:** Pearson's correlations between radiological markers of small vessel disease and quality of life subdomains

	Lacunes	CMB	WMH	SSQOL	ENE	LAN	MOB	MOD	PER	SC	SR	FAM	UEF	VIS	WOR	THI
Lacunes		**.46**	**.39**	**−.27**	−.07	**−.29**	**−.25**	**−.20**	**−.24**	**−.18**	**−.21**	**−.23**	**−.24**	−.05	**−.19**	**−.15**
CMB			**.50**	**−.21**	−.03	**−.27**	**−.16**	−.14	**−.21**	**−.18**	**−.19**	**−.18**	**−.23**	−.05	−.14	−.05
WMH				**−.16**	.02	−.15	−.13	−.15	−.09	−.14	−.13	−.14	**−.24**	−.02	−.08	−.13
SSQOL					**.71**	**.64**	**.76**	**.76**	**.74**	**.69**	**.81**	**.83**	**.74**	**.40**	**.80**	**.73**
ENE						**.35**	**.56**	**.49**	**.48**	**.37**	**.55**	**.53**	**.40**	**.25**	**.54**	**.54**
LAN							**.41**	**.56**	**.50**	**.33**	**.42**	**.45**	**.45**	**.17**	**.41**	**.55**
MOB								**.39**	**.37**	**.58**	**.53**	**.62**	**.66**	**.25**	**.70**	**.46**
MOD									**.68**	**.45**	**.57**	**.66**	**.40**	**.23**	**.48**	**.56**
PER										**.40**	**.58**	**.61**	**.40**	**.36**	**.50**	**.53**
SC											**.44**	**.57**	**.70**	**.28**	**.66**	**.32**
SR												**.66**	**.51**	**.28**	**.57**	**.59**
FAM													**.58**	**.26**	**.65**	**.55**
UEF														**.25**	**.69**	**.42**
VIS															**.35**	**.29**
WOR																**.51**
THI																

Note

Bold values are significant at *p*_FDR_ < .05.

Abbreviations: CMB, cerebral microbleeds; ENE, energy; FAM, family; LAN, language; MOB, mobility; MOD, mood; PER, personality; SC, self‐care; SR, social roles; SSQOL, Stroke‐Specific Quality of Life – Total Score; THI, thinking; UEF, upper extremity function; VIS, vision; WMH, white matter hyperintensities; WOR, work.

**TABLE 3:** Multivariate regression models with quality of life subdomains as the outcome variable

	Total SSQOL	Mobility	Language
Age	0.07 (0.79)	**−0.12 (0.006)**	0.02 (0.38)
Sex	2.75 (0.64)	0.23 (0.80)	0.08 (0.89)
NART	−0.03 (0.88)	−0.02 (0.53)	−0.02 (0.27)
Ethnicity			
Black	−5.45 (0.72)	0.55 (0.81)	−2.57 (0.066)
Caucasian	−7.75 (0.61)	−0.14 (0.95)	−2.52 (0.070)
Lacunes	**−8.22 (0.020)**	**−1.41 (0.008)**	**−0.69 (0.033)**
WMH	−2.47 (0.54)	−0.09 (0.87)	0.09 (0.81)
Microbleeds	−2.42 (0.37)	−0.41 (0.32)	−0.47 (0.056)

Note

Results are presented as unstandardized *β* (*p*).

As ethnicity is a categorical variable, it is tested at multiple levels.

Abbreviations: NART, national adult reading test; SSQOL, stroke‐specific quality of life; WMH, white matter hyperintensities.

Given that age was significantly associated with mobility QOL in multivariate models, we retested models with an exploratory age‐by‐lacune interaction term, although this yielded no significant results (Table S1).

### 3.3 Variables mediating relationships between lacunes and QOL

**FIGURE 1:**
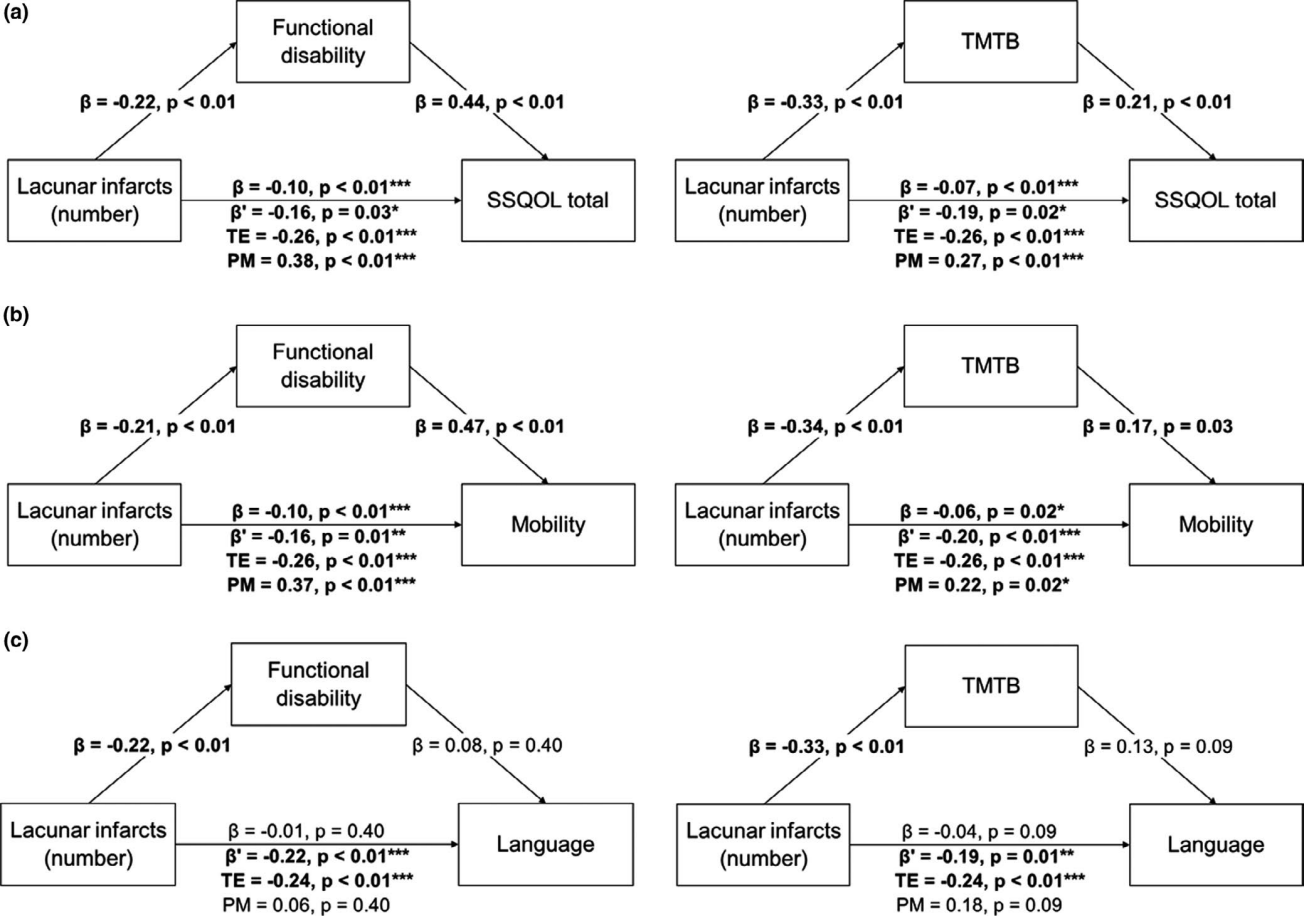
Models testing variables that may mediate the relationship between lacunes and quality of life. (a) Functional disability (left panel) and Trail Making Test‐B (TMTB; right panel) partially mediate associations between lacunes and total Stroke‐Specific Quality of Life (SSQOL) scale score. (b) Functional disability and TMTB partially mediate associations between lacunes and mobility‐related quality of life. (c) Functional disability and TMTB do not mediate associations between lacunes and language‐related quality of life. *β*, unadjusted coefficient; *β*′, coefficient after mediation; PM, proportion mediated; TE, total effect

Mediation models revealed that functional disability and TMTB partially mediated the relationship between lacunes and total SSQOL (Figure 1a). Functional disability and TMTB also partially mediated the relationship between lacunes and mobility (Figure 1b), but not between lacunes and language (Figure 1c). No significant partial mediation effect was found for VF (Figure S2). Notably, the direct effect of lacunes on QOL remained significant after controlling for mediating variables (*p* < .05).

## 4 DISCUSSION

Our study investigated relationships between radiological markers of SVD and specific subdomains of QOL in a pooled sample of symptomatic SVD patients. We found multivariate associations between lacune count and total QOL, as well as mobility and language subdomains. Associations with other SVD markers were not found, suggesting that lacunar infarcts are the most clinically detrimental marker of SVD. Furthermore, it suggests that each additional lacune decreases QOL in the mobility and language subdomains, possibly in a stepwise fashion. If this is the case, then it is possible that lacunes traditionally considered “asymptomatic” may still produce subtle clinical deficits.

We also attempted to determine the factors that may drive associations between pathology and QOL deficits. We found that functional disability and TMTB partially mediated associations between lacune count and total and mobility‐related QOL, but not language‐related QOL. VF did not mediate any QOL domains.

These mediation results suggest that distinct pathways lead from lacunar infarction to QOL deficits. Functional disability and executive function may partially explain relationships between lacunes and total and mobility‐related QOL deficits. Mobility‐related results suggest that patients are aware of the degree of functional impairment that they suffer from, which in turn has consequences on overall QOL. Executive function, on the other hand, also influences mobility‐related QOL, although the reasons behind this are unclear. Given associations between cognition and apathy in SVD (Tay, Lisiecka‐Ford, et al., 2020; Tay, Morris, et al., 2020), it is possible that a loss of motivation increases the perceived difficulty of performing everyday tasks, which is reflected in lower mobility‐related QOL. It should be noted that these effects were independent from age, which in itself was associated with mobility‐related QOL in multivariate models. This suggests that lacune‐related impairment compounds upon age‐related declines in mobility, which may have synergistic effects on QOL. Although we attempted to explore this possibility using an age‐by‐lacune interaction term, we did not find any significant effects, possibly due to models being underpowered given the number of included terms.

In contrast, neither functional disability nor executive function mediated relationships between lacunes and language‐related QOL. This was unexpected given that VF—which requires participants to produce words under a time limit—was also tested as a potential mediator, but no significant results were found. This suggests that impairment in the executive processes involved with producing and categorizing words has a minimal impact on language‐related QOL. These language deficits also appear to be unrelated to functional impairment. However, associations trending toward significance were seen for ethnicity. Complex associations between ethnicity, cardiovascular disease, and language have long been known (Pérez‐Stable et al., 1997), and it is possible that ethnicity could be an important mediating variable for the effect of lacunes on language‐related QOL. These relationships could be investigated in more detail in larger and more ethnically diverse cohorts.

Elucidating the pathways leading from specific markers of SVD and domain‐specific QOL deficits may lead to targeted treatment approaches. For instance, mobility‐specific QOL deficits could be explained by lacunes often occurring in subcortical structures involved in complex movement (Wardlaw et al., 2013), a hypothesis that can be tested in future investigations into the relationship between lacune location and QOL. Patients with radiologically confirmed lacunar infarcts could be recommended physiotherapy and prescribed exercise in order to potentially address these QOL deficits, even in the absence of overt movement difficulties.

Our study had limitations. One was that we could not include measures of apathy and depression in our analysis, given that the PRESERVE study did not assess these. Apathy may be an especially relevant mediating variable given its association with radiological markers of SVD and outcomes (Tay et al., 2019). Another limitation is that we could only include the patients in the SCANS and PRESERVE studies that had completed all of the included measures. This could have potentially excluded the most disabled patients, who would have difficulty completing all assessments. Finally, another limitation was that we did not examine QOL longitudinally. SVD pathology shows nonlinear associations with cognition over time (van Leijsen et al., 2019), and it is possible that this also applies to QOL.

In conclusion, we have shown that lacunar infarcts may have the most detrimental effects on QOL in a pooled sample of SVD patients from two cohorts. These deficits may be more pronounced in mobility and language‐related domains. Functional disability and executive function may partially mediate relationships between lacunes and mobility, but not language. These relationships hint at the potential for targeted treatment approaches that mitigate marker‐specific SVD‐related deficits, thereby improving overall QOL in patients.

## DISCLOSURES

None.

## CONFLICT OF INTERESTS

None declared.

## AUTHOR CONTRIBUTIONS

JF conducted the statistical analyses and wrote the manuscript. HE and ME were involved with the acquisition of the data. RBB, HSM, and JT conceived and designed the study. JF, RBB, HSM, and JT interpreted the data. All authors revised the manuscript critically for intellectual content.

### PEER REVIEW

The peer review history for this article is available at https://publons.com/publon/10.1002/brb3.2106.

[Correction added on March 20, 2021, after first online publication: Peer review history statement has been added.]

## Supporting information

Supplementary Material

## Data Availability

Anonymized data will be made available to qualified investigators upon reasonable request to the corresponding author.
