# Supplementary material for: Individual markers of cerebral small vessel disease and domain‐specific quality of life deficits
Source: Brain Behav. 2021 Mar 10;11(5):e02106. doi: 10.1002/brb3.2106 (PMC8119866; doi:10.1002/brb3.2106)
Supplement: Supplementary file 1 — Supplementary Material [file BRB3-11-e02106-s001.docx]

# Supplementary Figures


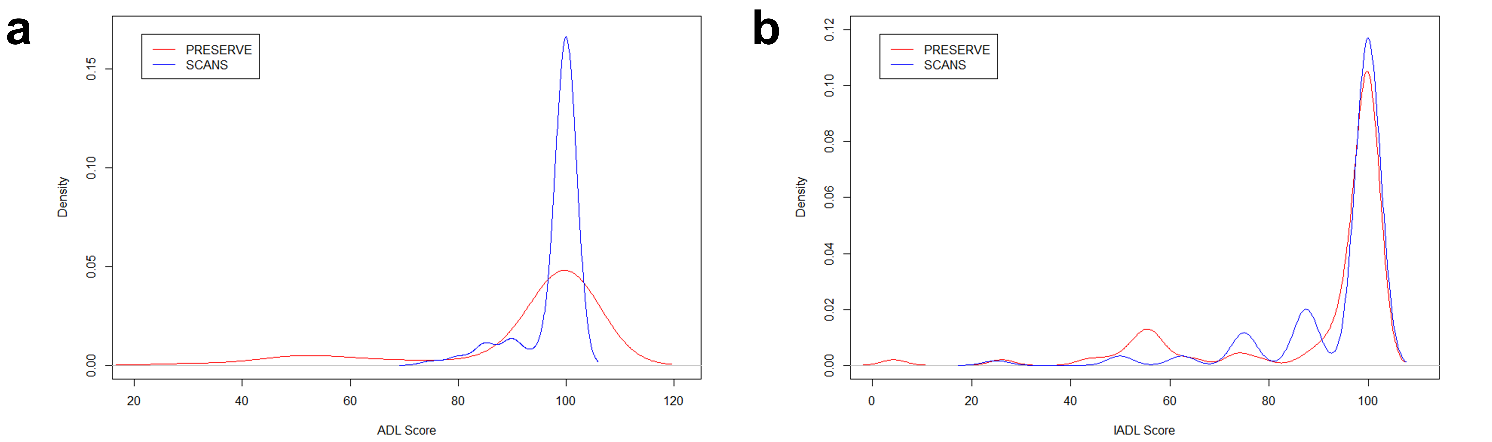


**Supplementary Figure 1.** Density plots of normalized distributions for activities of daily living scores. **a,** Basic activities of daily living assessed using the Barthel Index (SCANS) and Disability Assessment for Dementia Scale (PRESERVE). **b**, Instrumental activities of daily living assessed using the Instrumental Activities of Daily Living Scale (SCANS) and Disability Assessment for Dementia Scale (PRESERVE).


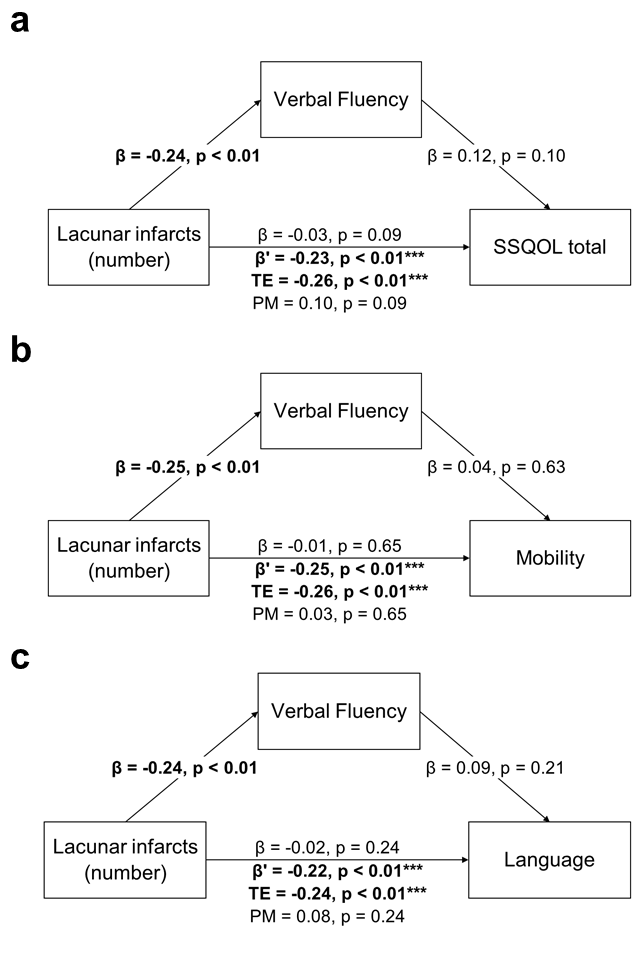


**Supplementary Figure 2.** Models testing the mediating effect of verbal fluency on the relationships between lacunes and quality of life. No mediation effects are found on the relationship between lacunes and **a,** total quality of life, **b,** mobility-related quality of life, and **c,** language-related quality of life.

# Supplementary Tables

**Supplementary Table 1.** Multivariate regression results with an age by lacunes interaction term.

|  | Total SSQOL | Mobility | Language |
| --- | --- | --- | --- |
| Age | -0.13 (0.80) | -0.11 (0.19) | 0.02 (0.76) |
| Sex | 2.32 (0.70) | 0.26 (0.78) | 0.06 (0.92) |
| NART | -0.05 (0.83) | -0.02 (0.56) | -0.02 (0.27) |
| Ethnicity |  |  |  |
| Black | -5.57 (0.71) | 0.56 (0.81) | -2.58 (0.07) |
| Caucasian | -7.73 (0.61) | -0.14 (0.95) | -2.52 (0.07) |
| Lacunar infarcts | -17.99 (0.39) | -0.78 (0.81) | -1.08 (0.57) |
| WMH | -2.32 (0.57) | -0.10 (0.87) | 0.10 (0.80) |
| Microbleeds | -2.32 (0.39) | -0.41 (0.31) | -0.47 (0.06) |
| Age-by-lacune interaction | 0.15 (0.63) | -0.0095 (0.84) | 0.0059 (0.84) |

*Note.* Results are presented as unstandardized β (P). SSQOL = Stroke Specific Quality of Life; NART = National Adult Reading Test; WMH = white matter hyperintensities. As ethnicity is a categorical variable, it is tested at multiple levels.
